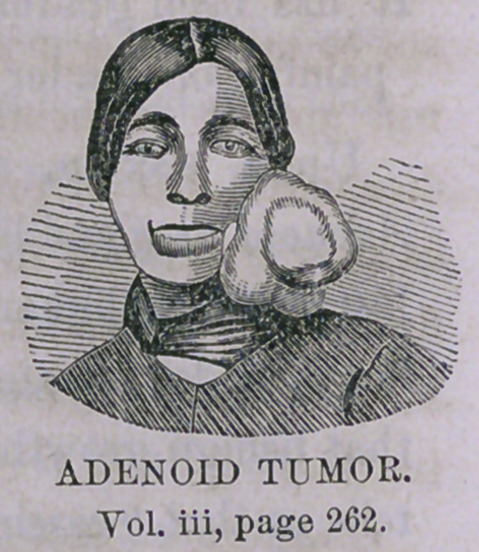# Clinical Remarks upon Surgical Cases in the Buffalo General Hospital—Removal of Fibrous Tumor—Removal of Adenoid Tumor—Strabismus—Talipes Valgus

**Published:** 1864-02

**Authors:** J. F. Miner


ART. V.—*Clinical Remarks upon Surgical Cases in the Buffalo General Hospital — Removal of Fibrous Tumor — Removal of Adenoid Tumor — Strabismus — Talipes Valgus.* By J. F. Miner, M. D.

November 21, 1863.

Gentlemen:—I am unable to report the results of our last operations, except that for excision of the eye. Immediately after the removal of the diseased eye, the other one, so much affected by sympathy, became free from all intolerance of light, and from the day after the operation the young man has been able to look up with distinctness and comfort; healthy granulations fill the space, and the case has left the hospital, "dismissed cured."

You observe that we have another large tumor presented for removal, attracted perhaps by the fame acquired in removing the one a few weeks since; the patients are from the same neighborhood, and have no doubt had communication. I would like to point out some differences, which are not obvious to the unprofessional, but which are important to you as surgeons. The first was the larger of the two, but it grew upon the back of the head and neck, a situation where no large arteries or nerves are located. It could then be removed without doubt and without risk. This one is situated over, or in, the most important surgical region—over, I hope, since I propose to attempt its removal. If I am mistaken, and it shall be found to extend too deeply for removal, the attempt is justifiable, and I shall proceed with care, cutting slowly and safely as possible. There are also differences in the character of these growths, which, though not very apparent previous to extirpation, will be doubtless sufficiently obvious after. Whatever be its nature, removal is desirable and proper. The important vessels situated in this region are quite familiar to you. The internal and external carotid arteries—internal and external jugular veins—pneumogastric and other important nerves.

Hemorrhage is the great fear of the young surgeon, which, after all, is not an accident most to be dreaded. Blood vessels can usually be ligated, and if serious hemorrhage is allowed during operations of this nature, it is more from want of firmness, and care, than from necessity. Division of nerves is often attended by serious results; paralysis of the muscles of the face, is not uncommon from the division of the facial nerve, while none of you need be told the results of division of the pneumogastric, presiding as it does over the functions of respiration and circulation. You can now observe that the tumor is covered by the integuments and platysma myoides muscle, upon the division of which we come down upon the cyst of the tumor, which is connected to the surrounding tissues quite strongly. These fibrous bands are carefully raised and divided, and the tumor is thus enucleated, and turned out with as little dissection as possible. It is supplied by large vessels, which my colleagues, Drs. Lothrop and Eastman, have ligated so immediately as to prevent serious hemorrhage. At the bottom of the cavity made by this growth you can see pulsating the internal carotid artery, and also that the tumor was over-lapped by the lower portion of parotid gland, and can judge from this, the importance of the surgical region, from which we have succeeded in safely removing this morbid product.

It appears upon section to have a strong fibrous cyst or covering, while the body of the tumor consists largely of fibrous tissue, in parts so dense as to resemble cartilage. It resembles in some respects malignant disease, but from its location and history I think it is benign in character. Microscopic examination might assist in the diagnosis, but the microscope does not always positively determine the character of these growths.

This tumor has been gradually increasing in size; was first noticed about fifteen years since. Has not caused much pain, but has been a deformity, and has also produced a constant feeling of fullness and discomfort. These unnatural and unhealthy products are quite common, and may make their appearance in any part of the system. Their causes are not known, though pathologists have a way of explaining their commencing growth, and possibly the accidents which determine their location. They are not benefited in any of their stages by medication, either general or local, unless we except the removal by caustic applications while small, and this is rarely, if ever, successful. The treatment we have adopted is the only rational one.

2*d Case.* Strange as it may be, we have also another tumor presented for removal, which would almost make it appear that the German population in Buffalo are made up largely of tumors. It is situated over the face, extending back to the ear, and below covering the angle of the lower jaw. It is lobulated in appearance, feeling as if made up of several distinct lobes or cysts; it rests firmly upon its base without much mobility and is supplied by numerous vessels; large dilated veins may be seen passing over its surface. It has been gradually increasing in size for the last twenty years; is not painful or tender on pressure; has not disturbed the general health.

Upon section, after removal, it appears to be what is denominated *Adenoid* or gland-like tumor. It had neither the appearance, symptoms or history of malignant disease, and has not been removed with any view that it would degenerate into, or take on such characters; indeed, it is probable that benign growths never degenerate into malignant disease. Rokitansky tells us that "carcinomata cannot, with adequate reason, be attributed to external local causes." However this may be, the growth removed has none of the characteristics of malignancy, and our patient may be assured that it is not likely to return.

We have had, then, for observation three different varieties of tumor.— The first removed, last week, was a large adipose or steatomatous growth, the second fibrous in character, and the third adenoid or gland-like. There are a great many different varieties of morbid growths which we denominate tumors, the first and most important division of which is into benign or simple, and malignant or cancerous; these are sub-divided, the benign into a great many different varieties. The principal points to be especially noticed, are first, the physical properties of the morbid growth; secondly, its relations to neighboring structures; and thirdly, you are to carefully study the history of all these morbid products, since it is important in forming correct diagnosis.

Tumors, it is believed, never change their original nature or degenerate into others of a different kind. A simple tumor never becomes malignant, nor does a malignant tumor ever become benign, though after the removal of simple tumors, malignant ones may make their appearance; but no simple tumor will by growth or degeneration become malignant; yet it is unquestionably true that the subject of simple tumor may also become the victim of malignant disease. This, I believe, is the correct position, though others are constantly expressing opposite views, and even the text-books are not uniform in their teachings upon these points. There are a great many other items of interest connected with the subject of tumors, which, however, it is no part of my purpose to discuss.

FIBROUS TUMOR.

Vol. iii, page 261.

ADIPOSE TUMOR.
Vol. iii, page 178.

ADENOID TUMOR.

Vol. iii, page 262.

*3d Case.—Convergent Strabismus.*—This being in a young boy, I propose to divide the tendon while he is under the influence of sulphuric ether. In children it is better to administer an anæsthetic before making this operation, while in adults it is usually pleasanter to make the operation without it. If no anæsthetic is given, the eye is more steady, and does not roll uncontrolled in every direction; it is under the influence of the will, and after division of the tendon it assumes its proper position, so that the operator is able to assure himself the better, that not only the tendon is divided, but also sufficient of the sub-conjunctival fascia and expansion of the tendon to allow the globe to assume a straight position. Ether embarrasses in this respect, while on every other account it is desirable. The opening through the conjunctiva should be made from before backwards, sufficiently to admit the raising of the tendon and its division, which is made mainly under the conjunctiva, sub-conjunctivally. In this way the deformity at the inner angle of the eye is greatly lessened.

It is a simple and easy operation to make, and remedies a greater deformity than any operation of equal importance in surgery. It has some dangers and some uncertainties of result, but it is almost uniformly successful and satisfactory. Successful in restoring symmetry in the axis of vision, but not in restoring vision itself. Satisfactory in appearance, but incapable of affording, in most cases, perfect sight.

You are all aware that we have convergent and divergent strabismus. It may be also congenital—existing at birth, or acquired—produced by disease or injury. In all cases, however, where motion is not lost—where there is not paralysis, division of the contracted tendon will remedy the deformity. You will be told much about the eye turning too far, or becoming more prominent than the other. Do not be deceived by these stories; divide the tendon and the eye will assume position to correspond with its fellow. The operation is followed with so uniformly favorable results, that I never feel any doubts as to the termination.

In the healing of the wound in the conjunctiva a small fungus is very apt to make its appearance; so common is this that you may expect that it will appear in nearly one-fourth of your cases. If left it will ultimately disappear; but it produces an unseemly appearance, some irritation, and usually gives rise to anxiety with the patient. It is often the size of a flattened pea, and it is always my habit to excise it with scissors; it is attached by a very slender pedicle, and may be removed without pain or trouble.

*Case 4th. Talipes Valgus* is the name given to the condition you observe in the foot of this young lady. There is not much arch of the instep, so that the sole of the foot is flattened, and there is eversion of the foot. The tendons of the peroneus longus, and brevis, behind the outer ankle are tense, and apparently too short, and so is that of the extensor communis on the dorsum. We divide sub-cutaneously these tendons and force the foot into a proper position. Strips of adhesive plaster are applied tightly, and over them a bandage so as to retain them and the foot in its new position.

Adhesive plaster, well applied, is the least expensive and most efficient apparatus for retaining the foot in desirable position. All machines fail but this, if applied in manner as now seen, will not fail. It is worth more than all other dressings. If you depend upon anybody's boots you are sure to fail in the results you desire to obtain. Many a well made operation for remedying this deformity is unsuccessful from neglect of this simple and only efficient dressing.

## Figures and Tables

**Figure f1:**
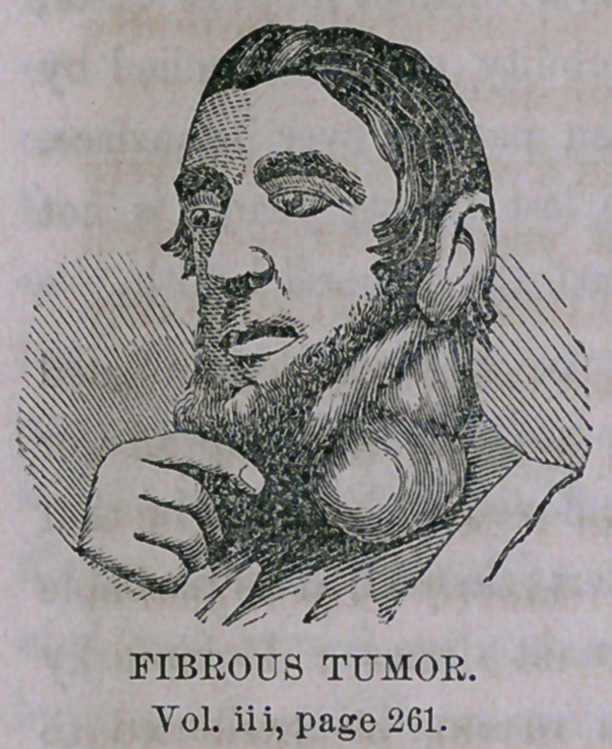


**Figure f2:**
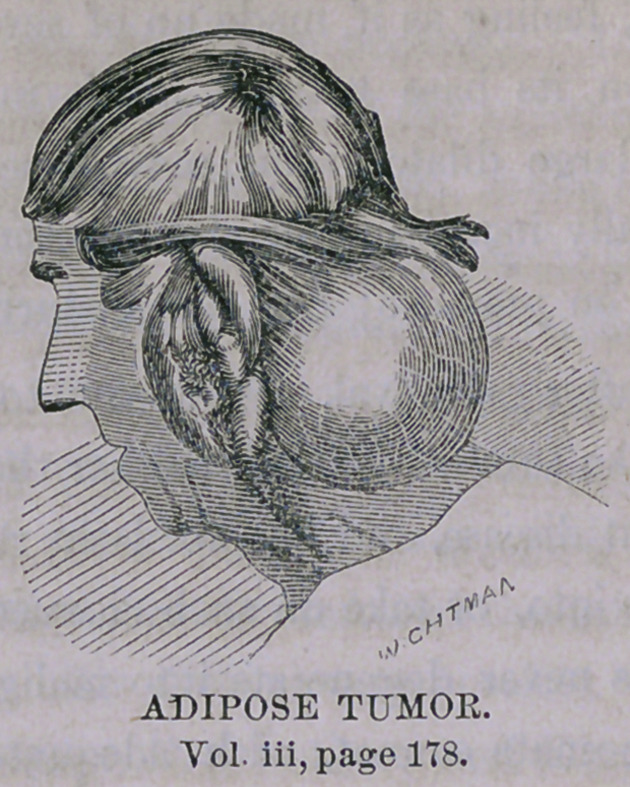


**Figure f3:**